# An overview of some enzymes from buthid scorpion venoms from Colombia: *Centruroides margaritatus, Tityus pachyurus,* and *Tityus* n. sp. aff. *metuendus*

**DOI:** 10.1590/1678-9199-JVATITD-2023-0063

**Published:** 2024-03-18

**Authors:** Leydy Lorena Mendoza-Tobar, Herlinda Clement, Iván Arenas, Juan Carlos Sepulveda-Arias, Jimmy Alexander Guerrero Vargas, Gerardo Corzo

**Affiliations:** 1 Grupo de Investigaciones Herpetológicas y Toxinológicas, Facultad de Ciencias Naturales, Exactas y de la Educación, Universidad del Cauca, Popayán, Colombia.; 2 Departamento de Medicina Molecular y Bioprocesos, Instituto de Biotecnología, Universidad Nacional Autónoma de México (UNAM), Cuernavaca Morelos, México.; 3 Grupo de Infección e Inmunidad, Facultad Ciencias de la Salud, Universidad Tecnológica de Pereira, Pereira, Colombia.

**Keywords:** Scorpion venom, Enzymes, Tityus, Centruroides, Phospholipase

## Abstract

**Background:**

In Colombia, several species of Buthidae scorpions belonging to the genera *Centruroides* and *Tityus* coexist, and their stings are considered life-threatening to humans because of their venom neurotoxins. Despite previous studies focusing on neurotoxins from these scorpion genera, little is known about the enzymes present in their venoms and their relationship with whole venom toxicity.

**Methods:**

Here, using proteomic and biochemical protocols the enzymatic activities of the venoms of three Colombian scorpion species, *C. margaritatus, T. pachyurus,* and *T.* n. sp. aff. *metuendus,* were compared to establish the presence and absence of enzymes such as phospholipases, hyaluronidases, and proteases that could be related to venom toxicity. **Results:** *C. margaritatus* was positive for hyaluronidases, *T.* n. sp. aff. *metuendus* for proteases, and *T. pachyurus* exhibited activity for all three mentioned enzymes.

**Conclusion:**

This information provides valuable insights into the specific enzyme diversity of each species’ venom and their potential role in venom toxicity, which could contribute to the development of better treatments and prevention strategies for scorpion envenomation.

## Background

Colombia is home to approximately 81 species of scorpions, including the genera *Tityus* and *Centruroides* of the Buthidae family such as *Centruroides margaritatus, Tityus pachyurus,* and *Tityus* n. sp. aff. *metuendus.* These species are known to cause scorpionism and significant histopathological damage, renal alterations, and cardiovascular effects, as demonstrated in recent studies [1- 8]. However, there has been limited research on the composition and biological activity of the different compounds in these scorpion venoms. Most studies to date have focused on characterizing the neurotoxins affecting potassium and sodium voltage-gated channels rather than the enzymatic composition [9- 15]. It is known that scorpion venoms are complex mixtures that include pharmacologically active components, including hydrolytic enzymes. Despite the limited knowledge of their role and composition, some studies have linked these types of enzymes in the venom to tissue permeability - acting as propagation factors, and to direct toxic effects in the development of some diseases of their victims [16- 18].

Although the enzymatic profile of scorpion venoms can vary based on the species studied, in general, phospholipases type A_2_ (PLA_2_), hyaluronidases, lysozymes, acetylcholinesterases, alkaline-phosphatases, metalloproteinases, and serine proteases have been identified. Some of these enzymes have not been described in terms of their function and have been established through transcriptome and/or proteomic work [17, 19- 22]. This research aims to carry out an enzymatic characterization of the venom of the three aforementioned scorpion species, providing relevant information on the toxicity of the venom and possible bioprospecting alternatives.

## Methods

### Scorpions

The collection of scorpions was carried out from different regions in Colombia based on their habitat. *Centruroides margaritatus* was collected from Valle del Patía and *Tityus* n. sp. aff. *metuendus* from Popayán, both sites in the Department of Cauca. *Tityus pachyurus* was collected from Ibagué, in the Department of Tolima. The collected scorpions were housed in well-ventilated wooden cages in the Universidad del Cauca animal facility and were provided food and water. The species *C. margaritatus*, *T. pachyurus,* and *T*. n. sp. aff *metuendus* were identified using dichotomous keys published on De Armas [4], Bohórquez-Gómez [23], and Guerrero-Vargas [24]. The venom was extracted through electrical stimulation, then collected in Eppendorf vials, lyophilized, and stored at -20 °C until use.

### Fractionation of scorpion venom by RP-HPLC

A portion of the venom extracted from the scorpions was fractionated using an RP-HPLC system and an analytical C_18_ reversed-phase column (Sigma-Supelco, Discovery C_18_, 4.6 x 250 mm, 5 μm). An aliquot of the scorpion venom containing 1.8 mg was resuspended in a distilled water solution and 0.1% TFA (referred to as solution A) and centrifuged to remove debris. Then, the soluble venom was chromatographed using a linear gradient that increased from 0 to 60% of solution B (acetonitrile + 0.1% TFA) over 70 min. The absorbance was measured at 230 and 280 nm, and the resulting fractions were collected manually, dried, and stored at -20 °C for later use [25].

### Determination of hyaluronidase activity

The hyaluronidase activity was determined for the whole and soluble venom of the three species (5 and 20 µg of the supernatant obtained after dilution and centrifugation). Fractions (2 and 3 µg) of the three study species were tested using a modified version of the protocol by Cevallos [26]. Briefly, a 12.5% acrylamide separation SDS-PAGE was prepared, incorporating hyaluronic acid as the substrate for hyaluronidases at a final concentration of 500 µg/mL. The samples were prepared in a non-reducing Laemmli buffer (without β-mercaptoethanol and heating), and 3 µg of *Brachypelma vagans* venom was used as a positive control. After electrophoresis, the gels were washed three times: the first wash was carried out with a 0.1 M phosphate buffer composed of glacial acetic acid and sodium acetate trihydrate at pH 3.6, enriched with 0.15 M sodium chloride and 5% Triton X-100 for two hours; the second wash was carried out with the same solution, plus 0.05% Triton X-100 for one hour; and finally, the third wash was carried out with acetate buffer for 10 minutes and left in a humid chamber overnight. Then, the gel was stained with 5 mL of a 0.1% Coomassie stock solution for five hours, and it was destained with a solution of 5% formamide, 20% isopropanol, and 0.015 M Tris-HCl buffer at pH 8. The representative positive bands of the fractions with the highest activity and different molecular sizes were selected, cut, and sent to the IBt-UNAM sequencing department for automatic Edman sequencing. Further amino acid sequence analysis was performed using the UniProt and NCBI database blast tools, and sequence alignment was performed using the Clustal Omega tool.

### Determination of proteolytic activity

The proteolytic activity for the whole and soluble venom of the three species was tested (5 and 20 µg of the supernatant obtained after dilution and centrifugation). Additionally, HPLC fractions (2 and 3 µg) of the three species studied were tested for protease activity. This was done by performing non-reducing electrophoresis using a modified version of the Laemmli protocol [27]. A 1.5 mg/mL acrylamide copolymerized gelatin separating gel was created. After electrophoresis, the gel was washed and incubated in a 50 mM Tris-HCl buffer solution at pH 8 with varying concentrations of Triton X-100, followed by overnight incubation in a humid chamber. The gel was then stained with Coomassie Brilliant Blue G-250 and destained with a 10% solution of acetic acid and 10% isopropanol. *Bothrops asper* venom was used as a positive control. The positive bands with the highest activity and different molecular sizes were selected, cut, and sent for sequencing through MALDI-TOF/MS sequencing at the IBt-UNAM sequencing department. Amino acid sequence analysis was performed using UniProt and NCBI database blast tools, and sequence alignment was done using the Clustal Omega tool.

### Determination of phospholipase activity

The phospholipase activity was determined for the whole venom (10 µg), the soluble venom (10 µg), and the HPLC fractions (2 - 3 µg). The protocol established by Habermann [28] was implemented with some modifications. Briefly, 0.2 g of agarose was dissolved in a 0.2 M Tris-HCl pH 8 solution. Then, 1 mL of 20 mM CaCl_2_, 2 mL of rhodamine, and 100 µL of Triton X-100 were added, followed by 2 mL of 10% egg yolk solution in a standard buffer (0.1 M Tris-HCl pH 8, 5 mM CaCl_2_, 8 mL of 0.1% rhodamine 6G and 100 µL Triton X-100). Then, the solution was poured into Petri dishes and allowed to solidify. Once solidified, small wells were created for the samples and controls; then, 10 µg of each sample was placed into the wells, with positive and negative controls placed at the ends. The plates were incubated for one hour at 37 °C and then observed under UV light to measure the halos resulting from the enzymatic activity. Distilled water or the buffer used for the egg solution preparation was used as a negative control, and 5 µg of *Micrurus fulvius* venom (1 µg/µL) was used as a positive control.

### Mass spectrometry and data analysis

The peptides resulting from trypsin digestion of the proteins separated in gel or proteins were purified utilizing a C_18_ resin microcolumn (ZipTip, Millipore), eluting directly with a matrix solution (3 mg/mL of alpha-cyano-4-hydroxycinnamic acid in 70% acetonitrile/0.1% TFA) on the MALDI plate in a volume of 1 μL. After co-crystallization on the plate, samples were analyzed by MALDI-TOF/TOF mass spectrometry for peptide fingerprinting (MS) on a mass spectrometer (4800 plus MALDI TOF/TOF Analyzer, ABSciex, Framingham MA) equipped with delayed extraction, reflective and in positive mode, in a mass/charge (m/z) range of 800 to 4,000 Da, with an accelerating voltage of 20 kV. Internal calibration of the spectra was performed using the mass/charge ratios (m/z) of the peptides resulting from the autolysis of porcine trypsin (M^+^H^+^ = 842.509, M^+^H^+^ = 2,211.104) obtaining a precision in the measurement of the m/z of ± 20 ppm. Fragmentation spectra (MS/MS) of the most intense m/z were obtained from each sample. Protein identification was performed by combining MS spectra and their corresponding MS/MS on public protein sequence databases (NCBI, UniProt), using MASCOT v2.0 (MatrixScience Ltd., London; http://www.matrixscience.com) or scorpion venom proteins in a fasta file using Protein Pilot v4.0 software (ABSciex; Framingham, MA, USA).

## Results and Discussion

### Separation of scorpion venoms using RP-HPLC

**Figure 1.**
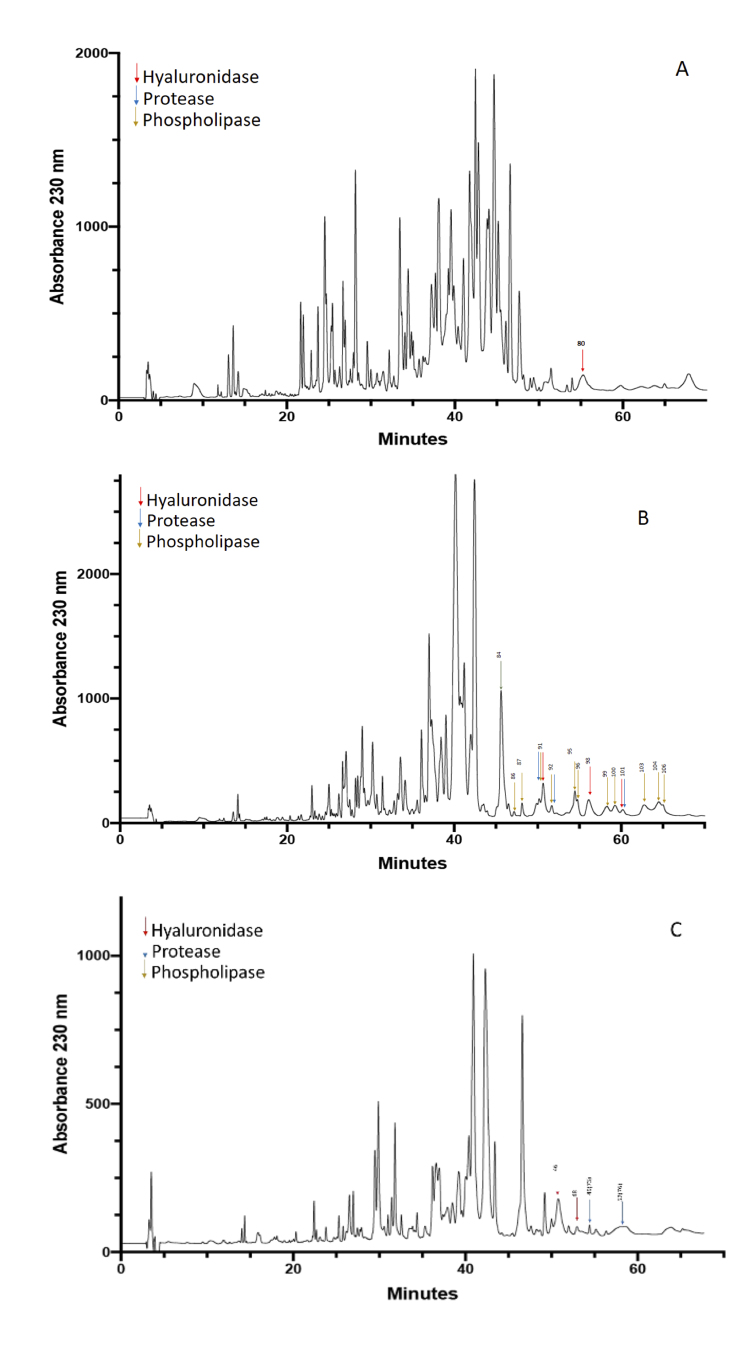
Separation profiles of the three venoms. **(A)** *Centruroides margaritatus*, **(B)** *Tityus pachyurus* and **(C)** *Tityus* n. sp. aff. *metuendus*. The arrows indicate the protein fraction numbers that were positive for hyaluronidases (red), proteases (blue), and phospholipases (yellow-brown).

The venom of the three species was separated into individual fractions using RP-HPLC, resulting in 85 fractions from *C. margaritatus* (Figure 1A), 106 fractions from *T. pachyurus* (Figure 1B), and 70 fractions from *T*. n. sp. aff. *metuendus* (Figure 1C).

### Hyaluronidase activities

Both the whole and soluble venoms from *C. margaritatus* and *T. pachyurus* showed hyaluronidase-positive results using zymogram assays, while similar assays for the venom from *T.* n. sp. aff *. metuendus* were negative (Additional file 1). After HPLC fractionation of the venom from *C. margaritatus*, the hyaluronidase activity of all protein fractions was tested. However, only fraction 80 was found to have positive activity (Figure 1A , red arrow). The protein band from fraction 80 with hyaluronidase activity from the venom of *C. margaritatus* was analyzed by MALDI-TOF/TOF (Additional file 2). The MS/MS sequencing results showed the presence of peptides related to the hyaluronidase protein family (Table 1). Further analysis using MASCOT showed coverage of 28% with the protein XP_023226974.1 (identity of 100 %, Table 2), which is a hyaluronidase 1-like sequence from the venom of *Centruroides sculpturatus,* and to the protein WDU65909.1 a putative hyaluronidase from the venom of *Tityus cisandinus* (identity of 81.7%, Table 2).

**Table 1.** Assignment of the RP-HPLC isolated fraction 80 of *C. margaritatus* venom to protein families, according to MALDI-TOF/TOF analysis of selected peptide ions from in-gel trypsin-digested protein bands.

Cm 80 peptide ions		Modifications	#PSMs	MS/MS-derived peptide sequence	Confidence	Ion Score	Protein family; related protein	Protein Accession*
m/z	z							
1062.11	3	2xCarbamidomethyl [C12; C19]; 1xOxidation [M5]	4	LAEDMRPDAGWCYYYFPDCYNYNGK	High	61	hyaluronidase 1-like [ *Centruroides sculpturatus*]	XP_023226974.1
857.96	2		6	DVMAPTIATVVLNTNR	High	109		
865.96	2	1xOxidation [M3]	10	DVMAPTIATVVLNTNR	High	91		
699.88	2		4	GDVNGGLLQVADLK	High	118		
757.87	2	1xCarbamidomethyl [C10]	4	VYWEVPSFLCSK	High	55		
644.35	2		1	DDITKFIPNPK	Medium	54		
921.46	3	1xCarbamidomethyl [C12]	2	LSWLWNQSTALCPSIYTQESHIK	High	23		
603.93	3	1xCarbamidomethyl [C3]	1	GHCYWPDEPFTSWK	High	25		

*NCBI Reference Sequence: XP_023226974.1

**Table 2.** Amino acid partial sequence of Cm 80 compared to a hyaluronidase from scorpion venom.

Protein*	Amino acid sequence
	1--------10--------20--------30--------40-------50--------60
Cm 80	---------------------- **VYWEVPSFLCSK**--------------------------
XP_023226974.1	MHSISIFSIFISIIYSAQADFK **VYWEVPSFLCSK**KYKINITQDLTSHKVLVNQGEGFNGD
WDU65909.1	MHLISIFSILISIIYAVQADFK **VYWEVPSFLCSK**KFKINVTQSLASHKVLVNQGEGFNGD
	61------70--------80--------90-------100------110--------120
Cm 80	-------------------- **GDVNGGLLQVADLK**------- **DDITKFIPNPK**--------
XP_023226974.1	KIVIFYENQLGKYPYIDPTK **GDVNGGLLQVADLK**EHLKVSK **DDITKFIPNPK**FDGIGVID
WDU65909.1	KIVIFYESQLGKYPYINSNDV **DVNGG**M **LQ**I **ADL**SEHLKVAK **D**N **ITKFIPNPK**FNGIGVID
	121-------130------140------150-------160------170-------180
Cm 80	----------------------------------------------------------- **L**
XP_023226974.1	WESWRPSWDFNWGKMKVYRERSIDLVKSKHPDWSSKKIEEAAIKEWEDSAKEWMLKTLK **L**
WDU65909.1	WEVWRPNWEFNWGKMKIYRQRSIDIVKSKHPTWPSNRIEEVAKEEWEKSAEEWMVKTMK **L**
	181-------190------200------210-------220------230-------240
Cm 80	**AEDMRPDAGWCYYYFPDCYNYNGK**------------------ **LSWLWNQSTALCPSIYTQ**
XP_023226974.1	**AEDMRPDAGWCYYYFPDCYNYNGK**DQPSQFTCNARVREQNSR **LSWLWNQSTALCPSIYTQ**
WDU65909.1	**A**Q **DMRPDA**A **WCYYIFPDCYNYNGK**DQAKQFACNPKIQAENSR **LSWLW**K **QSTA**I **C**H **SIY**M **Q**
	241-------250------260------270-------280------290-------300
Cm 80	**ESHIK**-------------------------------------------------------
XP_023226974.1	**ESHIK**KYNMSQRAWWIDARLRETMRVANPNTPIYPYINYVLPGTNETIPSMDFKRMLGQI
WDU65909.1	**ESHI**TKYNMTQRVWWTDARLREAIRVAHSNTPIYPYINYILPGENVKVVPAMDFKRMLGQ
	301-------310------320------330-------340------350-------360
Cm 80	-------------------------------- **DVMAPTIATVVLNTNR**--------- **GHC**
XP_023226974.1	IASLGLDGAIIWGSSYHVLTKSQCELTATYVK **DVMAPTIATVVLNTNR**CSQVICKGR **GHC**
WDU65909.1	IASLGLDGAIIWGSSYHVMTEPNCKITADYVN **DV**IS **PT**VA **TVVLNT**R **R**CSQVICKGR **G**N **C**
	361-------370------380------390--------402
Cm 80	**YWPDEPFTSWK**-------------------------------
XP_023226974.1	**YWPDEPFTSWK**YLIDPKLPVFKPTNISCKCKGYTGRYCQIAP
WDU65909.1	I **WP**E **EP**Y **TSWK**YLVDPKMPVFKPTNIYCRCKGYTGRYCQISQ

*NCBI Reference Sequence: XP_023226974.1 is a hyaluronidase 1-like from the venom of *Centruroides sculpturatus;* GenBank: WDU65909.1 putative hyaluronidase Tcis_Hyal1 from the venom of *Tityus cisandinus.*

Since the obtained results consist of small peptides rather than the complete amino acid sequence, it is not possible to conclude whether it represents a novel structure. However, based on the zymogram and MASCOT results, it can be said that the protein belongs to the family of scorpion hyaluronidases.

It is noteworthy that the discovered hyaluronidase has not been previously reported in the venom of *C. margaritatus*; however, similar enzymes have been found in other species of the genus *Centruroides*, such as *Centruroides edwardsii* from Costa Rica [29] and *Centruroides limpidus* from Mexico [30] *.* However, the zymogram of *Tityus pachyurus* venom fractions revealed several bands exhibiting hyaluronidase activity. Three bands of different molecular masses were selected and subjected to MALDI-TOF/TOF sequencing (Additional file 3). The MS/MS amino acid sequencing results of the protein band from fraction 91 confirmed the presence of peptides related to the hyaluronidase family (Table 3). Further analysis using MASCOT sequence analysis showed a 7% coverage and 100% identity with a putative hyaluronidase from *Tityus obscurus* venom from Brazil (JAT91136.1, Table 4). In addition, protein band 91 contained other peptide fragments belonging to the enzyme’s alpha-amidating peptidoglycan monooxygenase, alpha-amylase, and serine chymotrypsin protease.

**Table 3.** Assignment of the RP-HPLC isolated fractions 91 and 98 (98A and 98B) of *T. pachyurus* venom to protein families, according to MALDI-TOF/TOF analysis of selected peptide ions from in-gel trypsin-digested protein bands.

Peptide ions		Modifications	#PSMs	MS/MS-derived peptide sequence	Confidence	Ion Score	Protein family; related protein	Protein Accession*
m/z	z							
Tp 91							Putative hyaluronidase [ *Tityus obscurus*]	JAT91136.1
1060.84	3	1xCarbamidomethyl [C28]; 2xOxidation [M1; M23]	1	MLGQIASLGLDGAIIWGSSYHVMTEPNCK	low	47		
Tp 98A							Putative hyaluronidase [*Tityus obscurus*]	JAT91136.1
1060.84	3	1xCarbamidomethyl [C28]; 2xOxidation [M1; M23]	4	MLGQIASLGLDGAIIWGSSYHVMTEPNCK	High	78		
792.95	2	None	4	DVISPTVATVVLNTR	High	92		
648.86	2	None	3	IVIFYESQLGK	High	70		
1251.65	2	None	3	VAHSNTPIYPYINYILPGENVK	High	54		
1327.69	2	None	1	VLVNQGEGFNGDKIVIFYESQLGK	High	33		
883.89	2	1xCarbamidomethyl [C3]	2	GNCIWPEEPYTSWK	High	42		
757.87	2	1xCarbamidomethyl [C10]	2	VYWEVPSFLCSK	High	41		
898.14	4	2xCarbamidomethyl [C12; C19]; 1xOxidation [M5]	1	LAQDMRPDAAWCYYIFPDCYNYNGKDQAK	High	24		
Tp 98B		None					Putative hyaluronidase [ *Tityus obscurus*]	JAT91136.1
648.86		None	3	IVIFYESQLGK	High	70		
757.87		1xCarbamidomethyl [C10]	2	VYWEVPSFLCSK	High	41		
835.91		1xCarbamidomethyl [C10]	2	VYWEVPSFLCSKR	High	35		

*The putative hyaluronidase sequences from the venom of *T. obscurus* (GenBank: JAT91136.1) and from the venom of *Tityus bahiensis* (GenBank: JAG85181.1)

**Table 4.** Amino acid partial sequence of fractions 91, 98A, and 98B compared to a hyaluronidase from scorpion venom.

Protein*	Amino acid sequence
	1--------10--------20--------30--------40-------50--------60
Tp 91	------------------------------------------------------------
Tp 98A	---------------------- **VYWEVPSFLCSK**-------------- **VLVNQGEGFNGD**
Tp 98B	---------------------- **VYWEVPSFLCSK** *R*-------------------------
JAT91136.1	MHLISIFSILISIIYAVQADFK **VYWEVPSFLCSK**KFKINVTQSLASHK **VLVNQGEGFNGD**
	61------70--------80--------90-------100------110--------120
Tp 91	------------------------------------------------------------
Tp 98A	**KIVIFYESQLGK**------------------------------------------------
Tp 98B	- **IVIFYESQLGK**------------------------------------------------
JAT91136.1	**KIVIFYESQLGK**YPYINSNDVDVNGGMLQIADLSEHLKVAKDNITKFIPNPKFNGIGVID
	121-------130------140------150-------160------170-------180
Tp 91	------------------------------------------------------------
Tp 98A	----------------------------------------------------------- **L**
Tp 98B	------------------------------------------------------------
JAT91136.1	WEVWRPNWEFNWGKMKIYRQRSIDIVKSKHPTWPSNRIEEVAKEEWEKSAEEWMVKTMKL
	181-------190------200------210-------220------230-------240
Tp 91	------------------------------------------------------------
Tp 98A	**AQDMRPDAAWCYYIFPDCYNYNGKDQAK**--------------------------------
Tp 98B	------------------------------------------------------------
JAT91136.1	**AQDMRPDAAWCYYIFPDCYNYNGKDQAK**QFACNPKIQAENSRLSWLWKQSTAICHSIYMQ
	241-------250------260------270-------280------290-------300
Tp 91	-------------------------------------------------------- **MLGQ**
Tp 98A	------------------------- **VAHSNTPIYPYINYILPGENVK**--------- **MLGQ**
Tp 98B	------------------------------------------------------------
JAT91136.1	ESHITKYNMTQRVWWTDARLREAIR **VAHSNTPIYPYINYILPGENVK**VVPAMDFKR **MLGQ**
	301-------310------320------330-------340------350-------360
Tp 91	**IASLGLDGAIIWGSSYHVMTEPNCK**-----------------------------------
Tp 98A	**IASLGLDGAIIWGSSYHVMTEPNCK**------- **DVISPTVATVVLNTR**---------- **GNC**
Tp 98B	------------------------------------------------------------
JAT91136.1	**IASLGLDGAIIWGSSYHVMTEPNCK**ITADYVK **DVISPTVATVVLNTR**RCSQVICKGR **GNC**
	361-------370------380------390--------402
Tp 91	------------------------------------------
Tp 98A	**IWPEEPYTSWK**-------------------------------
Tp 98B	------------------------------------------
JAT91136.1	IWPEEPYTSWKYLVDPKMPVFKPTNIYCRCKGYTGRYCQISQ

*The putative hyaluronidase sequence from the venom of *T. obscurus* (GenBank: JAT91136.1) and from the venom of *Tityus bahiensis* (GenBank: JAG85181.1).

Furthermore, the sequencing result of the protein band from fraction 98 from *T. pachyurus* venom obtained via MALDI-TOF/TOF confirmed the presence of two proteins related to the hyaluronidases family (Table 3). Further analysis using MASCOT sequence analysis showed that a protein, named 98A, had a protein coverage of 36% and 98.6% identity with the putative hyaluronidase from the venom of the Brazilian *Tityus obscurus* (JAT91136.1) and another protein, named 98B, had a protein coverage of 6% and 87.5% identity with the putative hyaluronidase from the venom of *Tityus bahiensis* (accession 757180990) also from Brazil (Table 4).

Although fraction 101 presented a protein band with hyaluronidase activity, the results and discussion will be presented later.

The hyaluronidase activity zymogram conducted using the fractions of *Tityus* n. sp. aff. *metuendus* venom revealed two weak bands with apparent hyaluronidase activity, which were cut and sent for MS/MS amino acid sequencing. Out of the two extracted bands, only peptides from fragment 48 were obtained, which according to the sequencing analysis, does not correspond to a hyaluronidase, as seen in the whole venom zymogram. Until now, no hyaluronidase activity or partial sequence has been reported in the NCBI or UniProt databases for this scorpion species. However, hyaluronidase amino acid sequences have been reported for species of the same genus, such as *Tityus serrulatus*, *Tityus bahiensis, Tityus stigmurus*, *Tityus obscurus,* and *Centruroides sculturatus*, allowing us to reveal the identity of the enzymes studied in these three venoms. It is important to note that even though no positive results for hyaluronidase activity were obtained from the venom of *T*. n. sp. aff. *metuendus*, its presence within the venom cannot be ruled out. The enzyme’s concentration is possibly too low, or its activity is not strong enough to be detected; therefore, alternative methods for its determination should be considered. The significance of hyaluronidases in scorpion venom lies in their role as "dispersal factors." These enzymes hydrolyze hyaluronic acid (HA) in the interstitial matrix, enabling toxins in the venom to reach the victim’s bloodstream and invade the body. The enzymatic action of hyaluronidase increases the absorbance of the membrane and reduces its viscosity, making the tissues more permeable to the injected fluids, thus acting as a catalyst for systemic envenomation [31, 32]. Hyaluronidases are also commonly found in the venoms of various animals, such as spiders, snakes, and scorpions [33, 34], and serve as dispersing agents.

Moreover, Bordon et al. [35] showed that a hyaluronidase purified from the rattlesnake *Crotalus durissus terrificus* enhanced the toxicity of crotoxin, resulting in the death of animals in a co-injection group, while those injected with crotoxin alone survived the study [35]. In a recent study, a hyaluronidase isoform from the venom of the Peruvian snake *Bothrops atrox* was isolated and, when co-injected with the crude venom, increased the toxic effects as seen in the hemorrhage and hemolysis caused by the venom. However, hyaluronidase did not show any signs of toxicity [36]. In the case of scorpion hyaluronidases, their effect lies in the potentiation of toxicity. Studies by Horta et al *.* [37] showed that the inhibition and immunoneutralization of hyaluronidase reduced the toxic effects of *Tityus serrulatus* venom [37]. More recent studies in the same species showed that hyaluronidase has a crucial role not only in spreading the venom from the point of inoculation to the bloodstream but also in the biodistribution of the venom from the bloodstream to target organs, making this enzyme an important propagation factor and suggesting its inhibition as a potential first-aid strategy in case of poisoning [33]. In various fields of medicine, there have been reports of medical applications and unauthorized use of hyaluronidases [38, 39]. For example, cancer-derived cells often increase the expression of the CD44 receptor on the cell membrane, which acts as a receptor for hyaluronic acid and is associated with the migration, dissemination, attack, and metastasis of cancer-derived cells [40]. In light of this, a study showed that the BmHYA1 hyaluronidase isolated from the venom of the *Buthus martensii* scorpion reduced the expression of the CD44 variant in the breast cancer cell line MDA-MB-231 [41]. Another investigation reported that intranasal administration of hyaluronidase, either of bovine origin or isolated from *T. serrulatus* venom, reduced lung damage and fibrosis induced by bleomycin by recruiting mononuclear cells with phenotypic characteristics of mesenchymal stem cells toward the lung. This was associated with decreased lung damage and collagen deposition in the extracellular matrix, suggesting a potential treatment for pulmonary fibrosis [42].

### Protease activities

The zymogram performed to evaluate the proteolytic activity of the whole and soluble venom of *C. margaritatus* did not yield any positive results. This result was consistent with the findings from the *C. margaritatus* fraction analysis. The venom of this species, which was collected in Peru, was separated into nine fractions using a CM-Sephadex C-25 column, and none of the fractions displayed proteolytic activity [12]. On the other hand, the zymogram of the *T. pachyurus* fraction between 25 and 75 minutes showed two faint positive fractions with apparent proteolytic activity (fractions 91 and 92) (Figure 1B  blue arrows and Additional file 4). The bands were sent for MS/MS amino acid sequencing, and even though the blast of the peptide fragments of the sequencing results were positive for proteases, they were not of toxinological interest for our investigation because these enzymes were associated with posttranslational modifications such as peptidylglycine alpha-amidating monooxygenases [43]. Nevertheless, the product of the MS/MS amino acid sequencing of protein band 101, extracted from the hyaluronidase zymogram, was positive for a protease (Table 5). The sequencing result of band 101 from *T. pachyurus* obtained with MALDI-TOF/TOF confirmed the presence of a peptide related to proteases (Table 5). Further analysis using MASCOT sequence analysis shows a coverage of 3% and 100% identity with the metalloproteinase sequence of *T. obscurus* (JAT91159.1, Table 6), agreeing with the results described by Solano-Godoy et al. [44], where the presence of proteases in scorpion venoms was demonstrated.

**Table 5.** Assignment of the RP-HPLC isolated fraction 101 of *T. pachyurus* venom to protein families, according to MALDI-TOF/TOF analysis of selected peptide ions from in-gel trypsin-digested protein bands.

Tp 101 peptide ions		Modifications	#PSMs	MS/MS-derived peptide sequence	Confidence	Ion Score	Protein family; related protein	Protein Accession*
m/z	z							
627.89	2	None	3	NADIILLLITR	High	49	Putative metalloproteinase [ *Tityus obscurus*]	JAT91159.1

*GenBank: JAT91159.1

**Table 6.** Alignment of the sequence of fraction 101 (Tpachyurus_101_prot) extracted from the hyaluronidase zymogram of the *T. pachyurus* fractionated venom with the *T. obscurus* UNIPROT metalloproteinase sequence (A0A1E1WVV2,). The bold letters show the similarity between amino acid sequences.

Protein*	Amino acid sequence
	1--------10--------20--------30--------40-------50--------60
Tp 101	------------------------------------------------------------
JAT91159.1	MYLAYIFLFAAVSAIPTGRVEIVFPSVEQLRSGVKTVKFRALGEDVELKLEPAGDIIAKD
	61------70--------80--------90-------100------110--------120
Tp 101	------------------------------------------------------------
JAT91159.1	FAFYNGNHEKQQSMDIESLRKRLYRDRTNGAALLIDDDEQPPSIEGIVFSKLRISPHEWK
	121-------130------140------150-------160------170-------180
Tp 101	------------------------------------------------------------
JAT91159.1	EVTEDGKRAHQVEELTSDRDSYLYDNIILPDFQREMINFTRIERDDQCLVIEVLCVTEGN
	181-------190------200------210-------220------230-------240
Tp 101	------------------------------------------------------------
JAT91159.1	TERYETNEALTEYVTLMYSATETMLRQLDSGLQLRLSGIVAFTKETEPLFFKNIVHQDG
	241-------250------260------270-------280------290-------300
Tp 101	----------------------- **NADIILLLITR**--------------------------
JAT91159.1	DYKSGVLSEIRDYFCKNSTSLSK **NADIILLLITR**YMKLLKPNGSTRGIAQGIAYAGGVCD
	301-------310------320------330-------340------350-------360
Tp 101	------------------------------------------------------------
JAT91159.1	RCNKVNVIRGFMKPVSAAKTLAHEMAHLLGVPHDGKSSTGVSGSPGAKSCPSKDGFFMGD
	361-------370------380------390----398
Tp 101	--------------------------------------
JAT91159.1	NEGANWGIFSKCSKDCAKYLLSKPQASCVYEECKSSRY

*The putative metalloprotease sequence from the venom of *T. obscurus* (JAT91159.1).

For *T. pachyurus*, two fragments of two metalloproteinases encoded as V9ZAX6 and V9Z548 have already been reported in the UniProt database; however, there was no significant identity with the peptide fragment obtained in this work.

Regarding the evaluation carried out with the fractions of the *T*. n. sp. aff. *metuendus* venom, the positive results were observed slightly within fractions 73 and 76 (Figure 1C  blue arrows and Additional file 5). The bands were sent to the MS/MS amino acid sequence, but only one peptide fragment was obtained for protein band 76, confirming the presence of a scorpion protease (Table 7). 

**Table 7.** Assignment of the RP-HPLC isolated fraction 101 of *T. pachyurus* venom to protein families, according to MALDI-TOF/TOF analysis of selected peptide ions from in-gel trypsin-digested protein bands

Tp 76 peptide ions		Modifications	#PSMs	MS/MS-derived peptide sequence	Confidence	Ion Score	Protein family; related protein	Protein Accession
m/z	z							
495.80	2	None	1	**LIGIQAFTK**	High	49	Putative metalloproteinase [ *Tityus serrulatus*]	1214567054

GenBank: JAW07039.1

Further analysis using MASCOT sequence analysis showed coverage of 2% and 100% identity with the JAW07039.1 metalloproteinase sequence from the venom of *T. serrulatus* (Table 8) *.*

**Table 8.** Alignment of the amino acid sequence fragment of fraction 76 (Tspaffmetuendus_76_protease) extracted from the venom of *T.* n. sp. aff. *metuendus* with a metalloprotease from *T. serrulatus* (JAW07039.1). The bold letters show the similarity between amino acid sequences.

Protein*	Amino acid sequence
	1--------10--------20--------30--------40-------50--------60
Tp 76	------------------------------------------------------------
JAW07039.1	MIYFVSIFVFVTVSAIPTGREDVVFPWVETSRSGVKTVKFRALGEDIELKLEPAGDILAK
	61------70--------80--------90-------100------110--------120
Tp 76	------------------------------------------------------------
JAW07039.1	DFALLDLNNQRQPSVDVEKLRKRIYRDRVNGAALLIDDDESQSIEGIVNSKLRIAPHESR
	121-------130------140------150-------160------170-------180
Tp 76	------------------------------------------------------------
JAW07039.1	ELNQYGGRAHRIVELKSEKNSSLRDDVISRNIQRQIANFTSVSREDKCIVVEFLCVTESK
	181-------190------200------210-------220------230-------240
Tp 76	------------------------------------ **LIGIQAFTK**---------------
JAW07039.1	FTERFKTDQALTEYVTQMYTGVQNMYDTMNLEIKIR **LIGIQAFTK**ENEPSYIKESDVQNG
	241-------250------260------270-------280------290-------300
Tp 76	------------------------------------------------------------
JAW07039.1	KYILAGIIYKANNYYCKNATGLAQKADIIMLIVSRLLVWVKDSKITGNAVGIALGASACN
	301-------310------320------330-------340------350-------360
Tp 76	------------------------------------------------------------
JAW07039.1	KCEKVGVSLDETDYNERTITIAHEAGHMLGLPHDGQESTEVGVPNGPGAKSCPYDDGFIM
	361-------370------380------390------400
Tp 76	----------------------------------------
JAW07039.1	GSTIEPNMLKFSKCSKESAKYFFTLPQASCLREDCPNSGY

*The putative metalloprotease sequence from the venom of *T. serrulatus* (JAW07039.1). GenBank: JAW07039.1

Proteases are responsible for breaking down proteins into smaller fragments by cleaving them at specific sites, based on the amino acid sequence and the presence of specific amino acid residues at the N- or C- terminus or randomly. Proteases are categorized into three groups, based on the key amino acid (serine, cysteine, or aspartic acid) at the catalytic site or the need for a metal ion to perform their function (metalloproteases). These enzymes play a crucial role in cellular metabolism, for instance, by removing signal peptides and pro-peptides during the post-translational process. They can also function as toxins, which are well characterized in spider and snake venoms [45, 46]. Among the proteases described in venoms, metalloproteinases are the most common and are characterized by needing a bivalent ion as a cofactor to have their proteolytic activity [22, 47]. Metalloproteases such as anterases, which showed great similarity to proteases found in *T.* n. sp. aff *. metuendus,* are ubiquitous in a wide range of scorpion species, where they could be catalytically active enzymes. However, the proteolytic function in scorpions has not been very clear because they are enzymes commonly not detected in venom composition. In some studies, proteases in scorpion venoms were characterized [19, 20, 22, 48] and these proteases have been related to symptoms such as acute pancreatitis in species such as *T. serrulatus* because the anterase type proteases and specifically the Zn-metalloprotease found in this species cleave the proteins of the SNARE complex, which is responsible for pancreatic vesicular transport from the cytoplasm to the cell membrane [22, 49, 50].

### Characterization of phospholipase activity in the venoms of *Centruroides margaritatus*, *Tityus pachyurus*, and *Tityus* n. sp. aff. *metuendus*

For phospholipase characterization, an activity assay was initially performed using 10 µg of the whole venom (vt) and soluble venom (vs) from each species involved in this study (Additional file 6). Positive results were only obtained for *T. pachyurus*. The activity was proportional to the diameter of the observed halos, which indicated a phospholipase reaction. A halo of 1.7 cm was observed for the positive control (C+, *Micrurus fulvius* venom), followed by halos of 0.9 cm for the venom of *T. pachyurus*-vt and 0.7 cm for the venom of *T. pachyurus*-vs (Additional file 6). The difference between the latter two values, although they are from the same venom, might be related to the centrifugation process used to separate the soluble venom from the whole venom. Some of these enzymes may be lost within the pellet, which decreases their concentration relative to the uncentrifuged whole venom.

After chromatographic fractionation of the three venoms, a phospholipase activity test was performed. As a result, no positive reaction was observed in any of the fractions of the fractioned venom of *C. margaritatus*, which is consistent with the results obtained with the whole venom assay and with previous research conducted in Peru on the venom of individuals of this species [12]. Regarding the venom of *Tityus pachyurus*, thirteen of the tested fractions gave positive results (Figure 1C  and Additional file 7), with a phospholipase activity ranging from 0.5 to 0.8 cm compared to the positive control (C+), which had an average value of 1.6 cm. These results differ from those found by Solano-Godoy et al. [44]. This discrepancy is likely due to the low concentration of enzymes in the whole venom, making it not easily detected by the technique used in that study.

Only one previous report of enzyme activity has been found for this species, as mentioned previously. However, the presence of phospholipases in the venom of other species within the same genus, such as *T. serrulatus* and *T. obscurus*, has already been reported through proteomic studies. No positive reaction was observed in the venom fractions from *T*. n. sp. aff. *metuendus* or *C. margaritatus*, which is in line with the results obtained with the whole venom (Additional file 6).

Several types of venomous animals, including snakes, bees, and scorpions, secrete various phospholipases A_2_ (PLA_2_) that contribute both to the toxicity of the venom and to the digestion of the prey [51- 54]. The different PLA_2_s have been classified into four groups based on their primary structures: I, II, III, and IX [54]. They form a large family of enzymes characterized by low molecular weight (14-18 kDa), 5 to 8 disulfide bonds, a conserved His/Asp dyad with a histidine, and the requirement of calcium for catalytic activity [55]. The scorpion venom PLA_2_ belongs to group III 56] and is characterized by a particular heterodimeric structure composed of a long enzyme chain bound by a disulfide bridge to a short pentapeptide after the release of five residues during maturation [55]. Unlike snake venom with various svPLA_2_s identified, there are very few purified PLA_2_s in scorpion venom, including HfPLA_2_ from *Heterometrus fulvipes* [57], IpTxi and phospholipid from *Pandinus imperator*, Phaiodactylipin from *Anuroctonus phaiodactylus* [18], MtPLA2 from *Mesobuthus tamulus* [58], HmTx from *Heterometrus laoticus* [59], Hemilipins 1 and 2 from *Hemiscorpius lepturus* [60], Sm-PLGV from *Scorpio maurus* [61] *,* and *Centruroides hirsutipalpus* [48].

The structural models of the long chain of PLA_2_ from group III were constructed based on the crystal structure available from bee venom, which showed a high sequence identity. In all models, the general fold shows three α helices, an antiparallel two-stranded β sheet, and a calcium-binding loop. As in other svPLA_2_, a long N-terminal enzyme chain is coded during transcription, followed by a connecting pentapeptide and a short C-terminal extension. The long chain is stabilized by four disulfide bonds and is responsible for enzymatic activity. The short chain contains a free cysteine, which is suggested to form a disulfide bond with a free cysteine at the end of the long chain to covalently connect both parts even after the cleavage of the connecting pentapeptide. The function of this short chain is less clear, and its sequence is different in composition and length among different scorpion species. In some cases, it has a high content of hydrophobic residues and folds into an antiparallel β sheet, making it ideal for targeting specific tissues. 

The impact of pentapeptide insertions and the importance of short chains in enzyme activity were only addressed in a study of purified phospholipase A_2_ (Sm-PLGV) from the venom glands of the scorpion *Scorpio maurus* [53, 62]. This work allowed for the development of new potential agents against inflammatory protein targets or tumor angiogenesis. Additionally, other studies have succeeded in elucidating some biological activities of these enzymes, such as neurotoxicity and inflammatory effects. Numerous phospholipases A_2_ isolated from animal venoms are potent toxins that induce edema. The mechanism by which catalytically active PLA_2_ induces edema could be explained by the hydrolysis of phospholipids, probably due to the release of precursors of eicosanoids and platelet-activating factors. It is also important to note that catalytic activity has a key role, but it is not determinant in the edematogenic effect. In other words, phospholipase A2 can have a pharmacological domain that is independent of the catalytic site [63].

A study conducted with the phospholipase phaiodactylipin from *Anuroctonus phaiodactylus* showed a significant inflammatory effect when injected intramuscularly into the paw of mice. The enzyme causes significant edema by inflating the tissue. The cells are swollen but do not appear to cause damage to the basal membrane, as observed in similar preparations treated with purified phospholipases from snake venom [18]. However, there was no toxic effect when injected intraperitoneally.

Phospholipases have significant hemolytic activity. Studies seeking to clarify this activity indicate that kinetic studies of PLA_2_ in displacement mode establish that these enzymes bind to the surface of the membrane as a prelude to loading the active site with a single phospholipid molecule for the lipolysis reaction. It is becoming evident that sPLA_2_s from various sources can display different affinities for biomembranes composed of different groups of polar heads of phospholipids and fatty acid chains. This specificity of phospholipases has been widely used to explore the physical structure of phospholipids in biological membranes [64]. In this line, the discovery of new hemolytic PLA_2_s in venoms could help to understand the pathophysiology of envenoming, and also, to aid for developing experimental models of intravascular hemolysis. Among the scorpion venom PLA_2_s, only Phaiodactylipin, IpTxi, and Sm-PLGV display hemolytic activity toward erythrocytes [18, 62].

The native Sm-PLGV and its recombinant constructs rPLA_2_ (-5) and rPLA_2_ (+5) showed direct hemolytic activity despite their differences in specific activity. The hemolytic activity was tested with a suspension of human red blood cells. The long chain, which has lower activity than rPLA_2_ (+5) and rPLA_2_ (-5), showed the lowest hemolytic activity (10%) after the same incubation time. To confirm the relationship between the hemolytic and enzymatic activity, Sm-PLGV and recombinant variants were inactivated by the specific sPLA_2_ inhibitor, p-BPB. After total inhibition, all enzymes became unable to trigger the hemolytic effect. This result confirms the direct relationship between hemolysis and enzymatic activity [62].

Another activity of phospholipases is their anticoagulant activity. Phaiodactylipin, a phospholipase isolated from the venom of *Anuroctonus phaiodactylus*, and IpTxi from *Pandinus imperator*, both showed anticoagulant activity by increasing the coagulation time for both PPP (Platelet Poor Plasma) and human blood PRP (Platelet Rich Plasma). The normal control coagulation time is approximately 1 minute and 50 seconds. The coagulation time increased significantly by adding more than 5 μg of each enzyme. IpTxi is more effective than Phaiodactylipin, as concentrations above 10 μg confer noncoagulable properties to the blood, at least for a period of up to 30 minutes [18].

According to the mechanism proposed by Saikia [65], the anticoagulant effect of Phaiodactylipin and IpTxi may be related to enzymatic activity. These phospholipases hydrolyze procoagulant phospholipid PS and bind marginally to plasma phospholipids that are required in the coagulation process. It is well known that plasma phospholipids play a crucial role in the formation of several coagulation complexes. Therefore, the hydrolysis of PS (1-stearoyl-2-arachidonoyl-sn-glycero-3-[phospho-L-serine], which is known to be the most active phospholipid in the blood coagulation process) could lead us to anticipate the destruction of the phospholipid surface, which suggests as the main mechanism to explain the anticoagulant effect of this enzyme. This proposed mechanism is supported by Phaiodactylipin's ability to efficiently hydrolyze PS with a specific activity of approximately 63 U/mg [18].

According to the previously described mechanism, phospholipases not only assist in the diffusion of venom, but in some cases, they also present a toxic effect that can potentiate or cause the symptoms observed in venom-related intoxications, along with other toxins.

## Conclusion

Although we cannot affirm the total absence of certain enzymes in the studied species, such as in the case of phospholipase in *T*. n. sp. aff. *metuendus* and *C*. *margaritatus*, there is a relationship between the variety and number of enzymes detected with toxicity, as reflected in the LD_50_ reported in our previously published results, as well as in the envenoming reports, which show that the venom of *T. pachyurus* has caused more fatalities and severe clinical cases compared to *C. margaritatus* and *T*. n. sp. aff. *metuendus*. This coincides with the presence and diversity of enzymes in our investigation, as *T. pachyurus* showed three enzymes with sequence variability, while *C. margaritatus* only had one hyaluronidase, and *T*. n. sp. aff. *metuendus* only had one protease.

After obtaining fragments of the enzymatic sequences from the studied venoms and conducting analyses using tools such as MASCOT, as well as consulting databases such as NCBI and UniProt, the nature of the molecules containing these fragments was determined. However, no specific enzyme records were found for the studied species, constituting the first reports of these species regarding their enzymatic sequences. Furthermore, the high homology of these sequences with enzymes from other previously reported species indicates a possible common evolutionary origin and suggests structural or functional conservation over time. These findings highlight the importance of these enzymes in the biological context of the studied species and may open new lines of research regarding their role and potential applications.

### Abbreviations

SDS-PAGE, sodium dodecyl sulfate-polyacrylamide gel electrophoresis; TFA, trifluoroacetic acid.

## Supplementary material

The following online material is available for this article:

Additional file 1. Zymogram of hyaluronidase activity of the total and soluble venom of *Centruroides margaritatus, Tityus pachyurus* and *T. n. sp. aff. metuendus*. MM, protein markers; CmS, soluble venom of *Centruroides margaritatus*; CmT, total venom of *Centruroides margaritatus*; TpacS, soluble venom of *Tityus pachyurus*; TpacT, total venom of *Tityus pachyurus*; TpopS, soluble venom of *T. n. sp. aff. metuendus*; TpopT, total venom of *T. n. sp. aff. metuendus*; Positive control (C+), *Brachypelma vagans* venom. Stained with Stains-all 5 μg of each venom.

Additional file 2. Zymogram of hyaluronidase activity of *Centruroides margaritatus* venom fractions. MM *,* protein markers; lanes 74 to 84 *C. margaritatus* venom fractions; C+, positive control *Brachypelma vagans* venom. The red arrow indicates the positive fraction both in the chromatogram (Figure 1) and in the zymogram.

Additional file 3. Zymogram of hyaluronidase activity of *Tityus pachyurus* venom fractions. MM, protein markers. C+, positive control *Brachypelma vagans* venom *.* The red arrows indicate the positive fractions both in the chromatogram (Figure 1) and in the zymogram that were analyzed.

Additional file 4. Zymogram of protease activity of *Tityus pachyurus* venom fractions. MM, protein markers. C+, positive control *Bothrops asper* venom (1 μg). The red arrows indicate the positive fractions both in the chromatogram (Figure 1) and in the zymogram that were analyzed.

Additional file 5. Zymogram of protease activity of *Tityus* n. sp. aff. *metuendus* fractions. MM *, protein markers. C+, positive control* Bothrops asper venom (1 μg). Red arrows indicate the positive fractions both in the chromatogram (Figure 1) and in the zymogram that were analyzed.

Additional file 6. Determination of phospholipase activity of the venoms of *C. margaritatus, Tityus pachyurus,* and *Tityus n. sp. aff. metuendus*. C+, positive control *Micrurus fulvius* venom; CMT, *Centruroides margaritatus* total venom *;* CMS, *Centruroides margaritatus* soluble venom *;* TpacT *, Tityus pachyurus* total venom *;* TpacS *, Tityus pachyurus* soluble venom; TpopT, *Tityus n. sp. aff. metuendus* total venom *;* TpopS, *Tityus n. sp. aff. metuendus* soluble venom. C- *,* negative control PBS *.* The amount of venom was 10 µg of each *.*

Additional file 7. Determination of the phospholipase activity of *T. pachyurus* venom fractions. C+, positive control *Micrurus fulvius* venom; C- *,* negative control PBS. The amount of venom was 10 µg of each.
