# Supplementary material for: An overview of some enzymes from buthid scorpion venoms from Colombia: Centruroides margaritatus, Tityus pachyurus, and Tityus n. sp. aff. metuendus
Source: J Venom Anim Toxins Incl Trop Dis. 2024 Mar 18;30:e20230063. doi: 10.1590/1678-9199-JVATITD-2023-0063 (PMC10950367; doi:10.1590/1678-9199-JVATITD-2023-0063)
Supplement: Additional file 1. [file 1678-9199-jvatitd-30-e20230063-s1.pdf]

J Venom Anim Toxins incl Trop Dis
https://doi.org/10.1590/1678-9199-JVATITD-2023-0063

Supplementary Material to "An overview of some enzymes from buthid scorpion venoms from Colombia: *Centruroides margaritatus, Tityus pachyurus,* and *Tityus* n. sp. aff. *metuendus*"

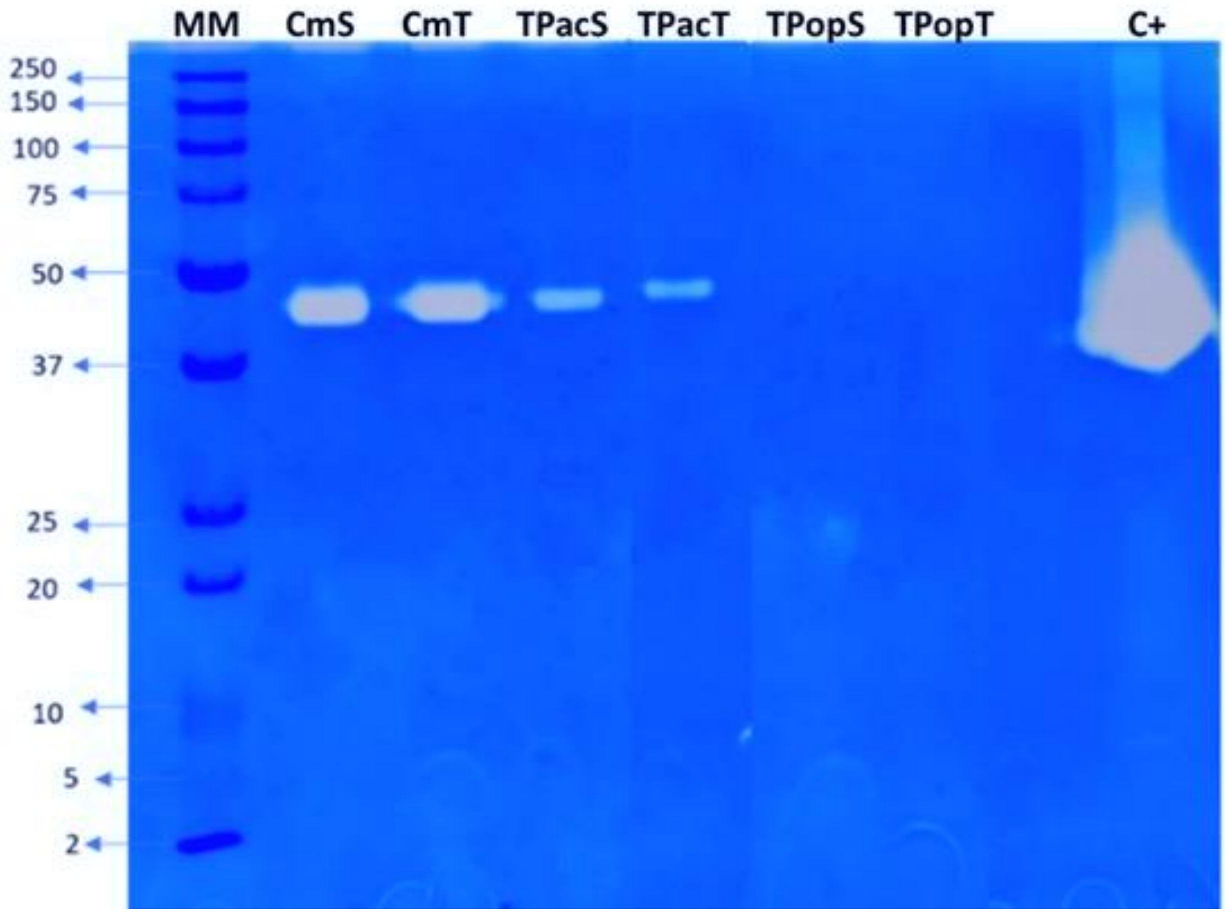

Additional file 1. Zymogram of hyaluronidase activity of the total and soluble venom of *Centruroides margaritatus*, *Tityus pachyurus* and *T.* n. sp. aff. *metuendus*. MM, protein markers; CmS, soluble venom of *Centruroides margaritatus*; CmT, total venom of *Centruroides margaritatus*; TpacS, soluble venom of *Tityus pachyurus*; TpacT, total venom of *Tityus pachyurus*; TpopS, soluble venom of *T.* n. sp. aff. *metuendus*; TpopT, total venom of *T.* n. sp. aff. *metuendus*; Positive control (C+), *Brachypelma vagans* venom. Stained with Stains-all 5 µg of each venom.
