# Supplementary material for: An overview of some enzymes from buthid scorpion venoms from Colombia: Centruroides margaritatus, Tityus pachyurus, and Tityus n. sp. aff. metuendus
Source: J Venom Anim Toxins Incl Trop Dis. 2024 Mar 18;30:e20230063. doi: 10.1590/1678-9199-JVATITD-2023-0063 (PMC10950367; doi:10.1590/1678-9199-JVATITD-2023-0063)
Supplement: Additional file 2. [file 1678-9199-jvatitd-30-e20230063-s2.pdf]

J Venom Anim Toxins incl Trop Dis
https://doi.org/10.1590/1678-9199-JVATITD-2023-0063

Supplementary Material to "An overview of some enzymes from buthid scorpion venoms from Colombia: *Centruroides margaritatus, Tityus pachyurus,* and *Tityus* n. sp. aff. *metuendus*"

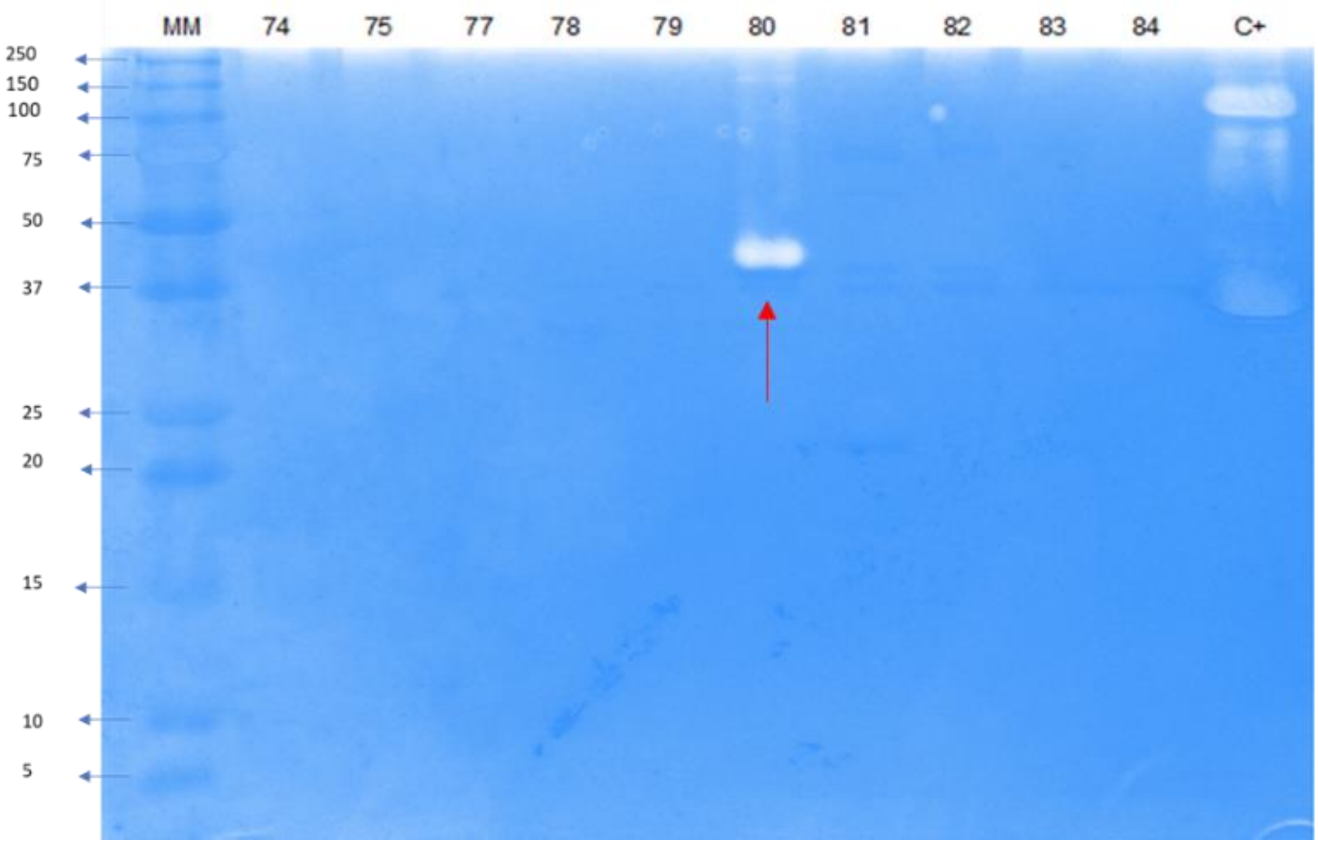

Additional file 2. Zymogram of hyaluronidase activity of *Centruroides margaritatus* venom fractions. MM, protein markers; lanes 74 to 84 *C. margaritatus* venom fractions; C+, positive control *Brachypelma vagans* venom. The red arrow indicates the positive fraction in the chromatogram (Figure 1) and in the zymogram.
