# Supplementary material for: An overview of some enzymes from buthid scorpion venoms from Colombia: Centruroides margaritatus, Tityus pachyurus, and Tityus n. sp. aff. metuendus
Source: J Venom Anim Toxins Incl Trop Dis. 2024 Mar 18;30:e20230063. doi: 10.1590/1678-9199-JVATITD-2023-0063 (PMC10950367; doi:10.1590/1678-9199-JVATITD-2023-0063)
Supplement: Additional file 5. [file 1678-9199-jvatitd-30-e20230063-s5.pdf]

J Venom Anim Toxins incl Trop Dis
https://doi.org/10.1590/1678-9199-JVATITD-2023-0063

Supplementary Material to "An overview of some enzymes from buthid scorpion venoms from Colombia: *Centruroides margaritatus, Tityus pachyurus,* and *Tityus* n. sp. aff. *metuendus*"

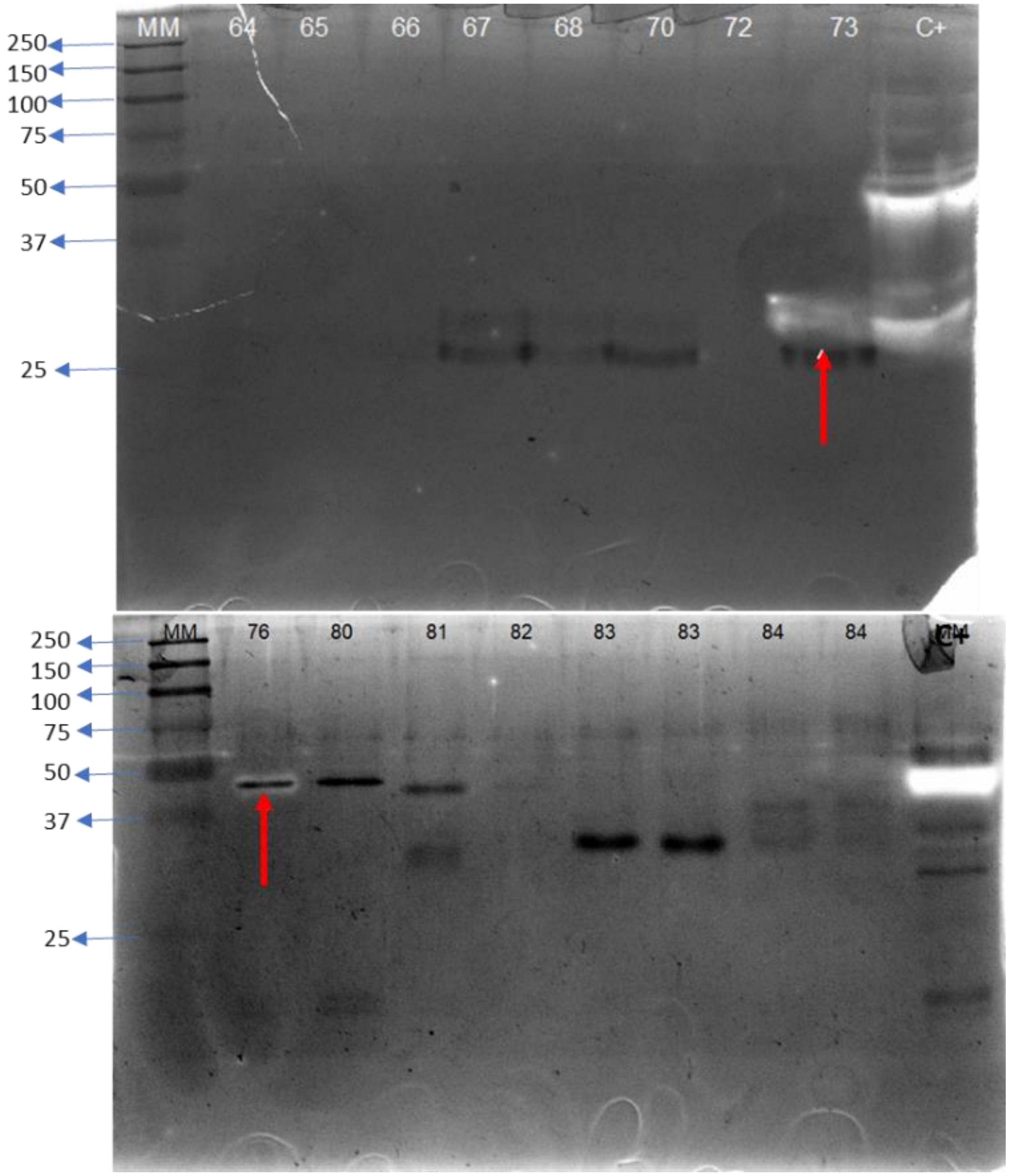

Additional file 5. Zymogram of protease activity of *Tityus* n. sp. aff. *metuendus* fractions. MM, protein markers. C+, positive control *Bothrops asper* venom (1 µg). Red arrows indicate the positive fractions both in the chromatogram (Figure 1) and in the zymogram that were analyzed.
