# Supplementary material for: An overview of some enzymes from buthid scorpion venoms from Colombia: Centruroides margaritatus, Tityus pachyurus, and Tityus n. sp. aff. metuendus
Source: J Venom Anim Toxins Incl Trop Dis. 2024 Mar 18;30:e20230063. doi: 10.1590/1678-9199-JVATITD-2023-0063 (PMC10950367; doi:10.1590/1678-9199-JVATITD-2023-0063)
Supplement: Additional file 6. [file 1678-9199-jvatitd-30-e20230063-s6.pdf]

J Venom Anim Toxins incl Trop Dis
https://doi.org/10.1590/1678-9199-JVATITD-2023-0063

Supplementary Material to "An overview of some enzymes from buthid scorpion venoms from Colombia: *Centruroides margaritatus, Tityus pachyurus,* and *Tityus* n. sp. aff. *metuendus*"

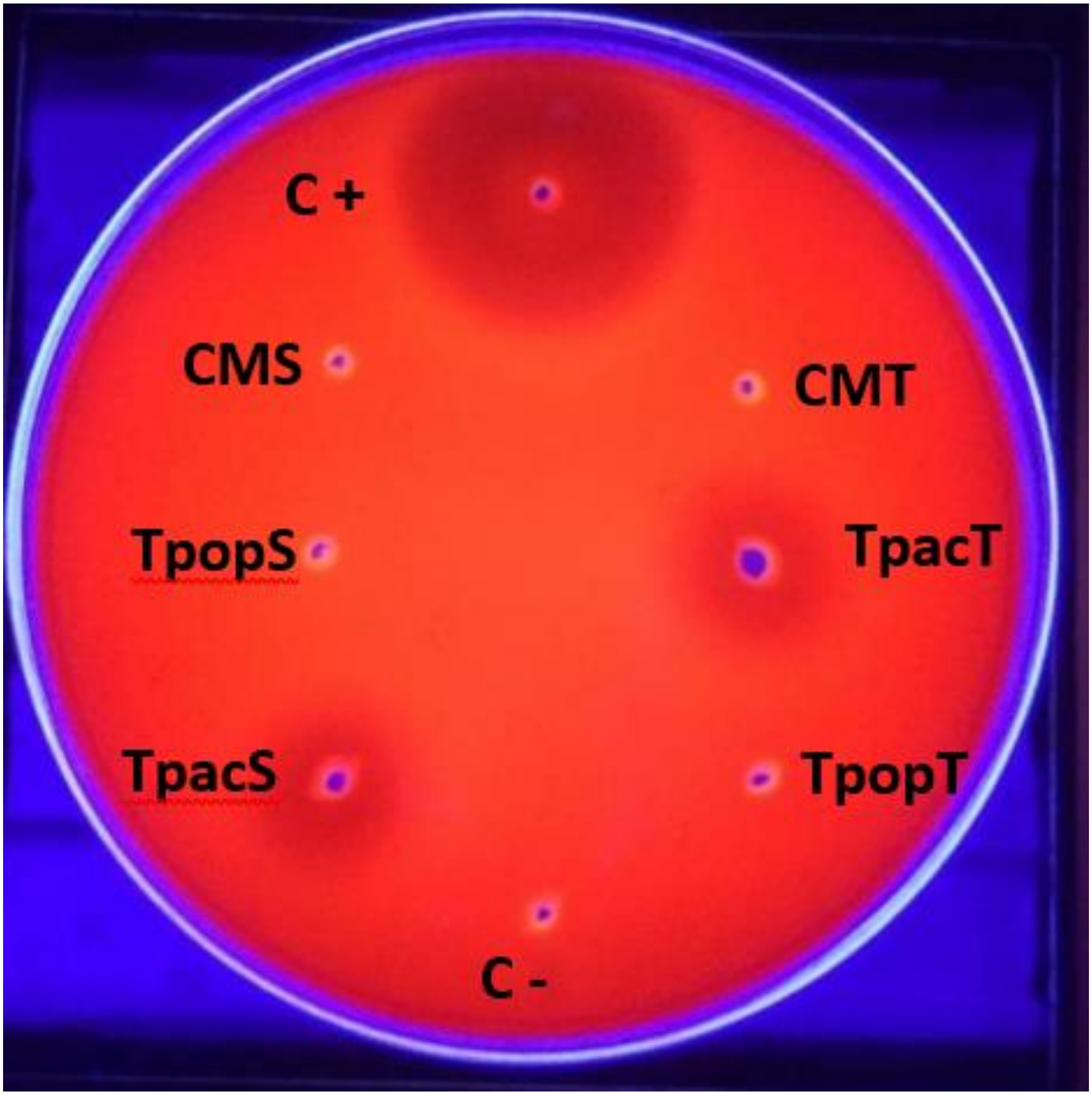

Additional file 6. Determination of phospholipase activity of the venoms of *C. margaritatus*, *Tityus pachyurus*, and *Tityus* n. sp. aff. *metuendus*. C+, positive control *Micrurus fulvius* venom; CMT, *Centruroides margaritatus* total venom; CMS, *Centruroides margaritatus* soluble venom; TpacT, *Tityus pachyurus* total venom; TpacS, *Tityus pachyurus* soluble venom; TpopT, *Tityus* n. sp. aff. *metuendus* total venom; TpopS, *Tityus* n. sp. aff. *metuendus* soluble venom. C-, negative control PBS. The amount of venom was 10 µg of each.
